# Phytochemical analysis of *Stachys iva*: Discovering the optimal extract conditions and its bioactive compounds

**DOI:** 10.1515/biol-2022-1053

**Published:** 2025-02-19

**Authors:** Aikaterina Vantsioti, Ioannis Makrygiannis, Vassilis Athanasiadis, Stavros I. Lalas, Paraskevi Mitlianga

**Affiliations:** Department of Chemical Engineering, University of Western Macedonia, 50100, Kozani, Greece; Department of Food Science & Nutrition, University of Thessaly, Terma N. Temponera Str., 43100, Karditsa, Greece

**Keywords:** *Stachys*, optimization, polyphenols, antioxidant, anti-inflammatory

## Abstract

The Lamiaceae family is one of the widest plant families among Greek flora, consisting of a great variety of species, with the genus *Stachys* being one of its largest representatives, spread to most continents. The genus *Stachys* is also known for its beneficial properties and has been used for years as a traditional remedy for healing various health conditions. *Stachys iva*, an endemic plant in the Kozani Regional unit, has also been consumed as an infusion by locals and is reported to relieve common cold symptoms, have antimicrobial properties, and contribute to normalizing blood glucose levels. The present study aimed to identify the chemical compounds (such as phenolic acids, flavonoids, and phenylethanoid glycosides) responsible for the herb’s pharmacological properties and determine the optimal extraction conditions to gather an extract with high therapeutic value without solvent and energy waste. Experiments conducted proved that extracting by simple stirring with deionized water for 75 min at 80°C is the best option. In contrast, the extract’s total polyphenol content was determined, and the compounds were identified by high-performance liquid chromatography analysis. In addition, other methods were utilized (e.g., ferric-reducing antioxidant power assay and 2,2-diphenyl-1-picrylhydrazyl antiradical activity assay) to reveal potent antioxidant, anti-hydrogen peroxide, and anti-inflammatory activity, while the correlation between these properties and extraction conditions was also examined.

## Graphical Abstract

**Figure:**
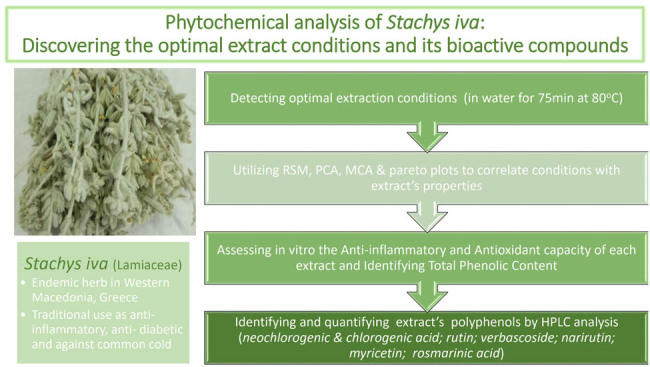

## 1 Introduction

Genera of the Lamiaceae family are widely known for their therapeutic properties and the variety of traditional uses as remedies over the ages. Since ancient times, different Lamiaceae species, conditioned as herbal infusions, edible greens, and extracts, have been mostly consumed as antioxidant, anti-inflammatory, antimicrobial, and analgesic herbal medicines [1,2]. Specific examples of genera from the Lamiaceae family known for their therapeutic properties include *Lavandula* (lavender) for its calming effects, *Mentha* (mint) for digestive health, and *Salvia* (sage) for its anti-inflammatory properties. All the above properties are also common among the species of genus *Stachys*, one of the largest representatives of the Lamiaceae family as it includes more than 300 species, spread around the Mediterranean area, Asia, America, and southern Africa [3]. The phytochemical analysis conducted on several *Stachys* species has scientifically verified the empirical use of these herbs as a treatment for different conditions. The polyphenolic compounds which have been isolated and identified are related to the inhibition of lipid peroxidation and result in antioxidant and anti-inflammatory activity [4,5,6]. In addition, flavonoids, iridoids, and glucosides seem to enhance the anti-inflammatory activity. Fatty acids, alkaloids, triterpenes, lignans, and secondary metabolites have also been isolated from different *Stachys* species [2,7,8]. The numerous pharmacological activities of the latter are also significant as – except for those mentioned above – some species have been efficiently used as antibacterial agents [9,10,11,12], as well as treatment for polycystic ovary syndrome and as potent hepatoprotective, as well as neuroprotective agents against Alzheimer’s disease. Extracts have been additionally examined for their anti-proliferative cytotoxic and antidiabetic properties, proving once again the plethora of pharmacological activities as a potent alternative approach to diabetes and cancer treatment [2,3,5,8,10,13,14].

Despite the plethora of *Stachys’* beneficial properties, the potential side effects or even the possibility of interaction among its extracts and other medications should be considered before consuming its infusions. Even though there is not adequate information about *Stachys iva* toxicity or contraindications – as it has not been thoroughly studied before – some *Stachys* species are mentioned to be emmenagogue and uterotonic while others cause hypotension [15] or even are toxic damaging liver and renal tissue [16]. In any case, as the above data are derived from experiments in rats, pregnant women and individuals with chronic health issues consuming medicaments should not better consume any herbal remedies without a specialist’s advice due to lack of evidence about potential adverse effects.

Polyphenolic compounds found in abundance in *Stachys* extracts are more likely to be responsible for their therapeutic properties. These compounds can be found in many fruits, vegetables, and plants, being associated with their beneficial properties and some of their characteristics such as their color, flavor, and odor [17]. Chemically, they contain phenolic rings combined with various structures which are responsible for their classification as well as for their properties. Phenolic acids, flavonoids, lignans, and stilbenes are the main groups of chemical compounds classified as polyphenols [18].

Several studies have been conducted to determine how polyphenol consumption improves our health [19,20]. Primarily, polyphenols are considered to be a natural “weapon” against inflammation. Even inflammation is a natural response of our immune system after tissue damage or infection as an attempt to restore the primer healthy condition, it is characterized by unpleasant symptoms such as redness, pain, swelling, and heat leading, if not suppressed on time, to loss of tissues or even organ’s function. Since the pathophysiology of inflammation is complicated, involving immune cell responses (mainly by cytokines) and a plethora of biochemical molecules that trigger the procedure (such as prostaglandins, cyclooxygenase, and TNFa), its suppression requires a kind of intervention in this “mechanism” [21].

Polyphenolic compounds are also characterized as natural antioxidants, providing oxidative stability to the plant and scavenging free radicals when consumed [17,20]. Even though reactive oxygen species (ROS) are necessary for signal transmission involved in crucial biological functions, their excessive production results in bonding with macromolecules, causing DNA damage, and mutations and affecting normal functions in general [22]. As a result, chronic exposure to oxidative stress due to free radical formation is the main “suspect” for causing cancer, inflammation, as well as degenerative and circulatory system conditions [23,24,25].

**Figure 1:**
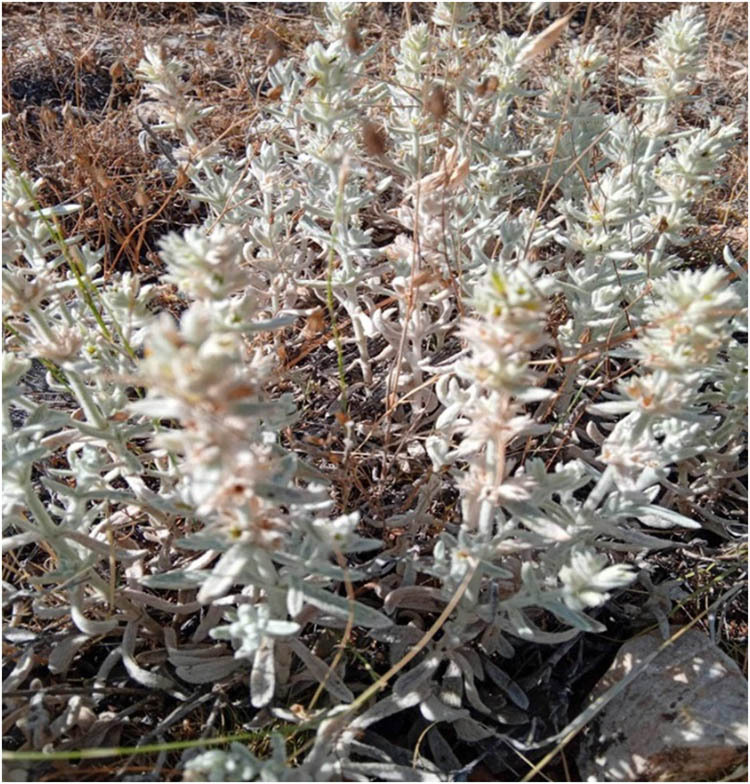
A typical *Stachys iva* plant.

Summarizing, polyphenols seem to have a protective role in inflammatory (chronic) conditions, neurodegenerative diseases, and many other consequences of aging in general, whereas several epidemiological studies have co-related the polyphenolic compounds consumption with the reduction of coronary artery disease risk and glucose levels in the blood as well [26]. Even though *Stachys* species have been thoroughly studied, some endemic species such as *Stachys iva* are still “unexplored.” *S. iva*, known as “fluffy mountain tea,” grows in Northern Greece, especially in the mountains of Kozani Regional unit (Western Macedonia), at an altitude of approximately 600 m [27]. It is a perennial herb with a hairy four-angled stem and opposite narrow leaves. Flowers occur in summer, and the corollas are yellow, sympetalous, and two-lipped [28]. A typical *S. iva* plant with its leaves is illustrated in Figure 1.

As *S. iva* has been traditionally used as a herbal infusion instead of *Sideritis* spp. due to its antibacterial properties as well as a remedy for the relief of common cold symptoms, the present study aims to identify the chemical compounds that could be responsible for these therapeutic properties. Regarding the identification of the active compounds, high-performance liquid chromatography (HPLC) has been utilized, combined with other conventional experimental procedures. Specifically, Folin–Ciocalteu and ferric-reducing antioxidant power (FRAP) assay, in addition to 2,2-diphenyl-1-picrylhydrazyl (DPPH) antiradical activity assay and hydrogen peroxide scavenging assay, were conducted for the determination of the total phenolic compounds and antioxidant/antiradical capacity of the extract. The anti-inflammatory activity of the *S. iva* extract has also been assessed *in vitro* as an attempt to experimentally justify some of the plant’s pharmacological properties.

An additional purpose of this study is to detect the optimal extraction conditions of *S. iva* in order to investigate which temperature–duration combination leads to the most enriched, in bioactive compounds, extract. The optimization also has an environmentally friendly aspect, as it aims to the reduction of energy and solvent consumption. Moreover, using water as solvent through the process does not involve harmful organic solvents which are harmful to the environment and may provide extracts unsuitable for consumption by humans. To the best of our knowledge, there are no other comprehensive phytochemical analyses of *S. iva* available in the literature. Therefore, our study represents a novel contribution to the understanding of this species’ bioactive compounds.

## 2 Materials and methods

### 2.1 Chemicals and reagents

All the experiments were conducted by using mainly deionized water as solvent except for solvents used in chromatography (water, formic acid, and acetonitrile) that were HPLC grade. Neochlorogenic acid, chlorogenic acid, rutin, verbascoside, narirutin, myricetin, and rosmarinic acid were purchased from Sigma-Aldrich (Darmstadt, Germany) as chemical standards and used in HPLC for determination of polyphenolic compounds. Deionized water was chosen as the extraction solvent due to its safety, cost-effectiveness, and ability to extract a wide range of polyphenolic compounds.

Hydrochloric acid, phosphate buffer solution, methanol, l-ascorbic acid, trichloroacetic acid, aluminum chloride, 2,4,6-tris(2-pyridyl)-s-triazine (TPTZ), and 2,2-diphenyl-1-picrylhydrazyl (DPPH) were also purchased from Sigma-Aldrich (Darmstadt, Germany). The Folin–Ciocalteu reagent, ethanol, and gallic acid were obtained from Panreac Co. (Barcelona, Spain). Hydrogen peroxide (35% v/v) was purchased from Chemco (Malsch, Germany), iron(iii) chloride from Merck (Darmstadt, Germany), and anhydrous sodium carbonate from Penta (Prague, Czech Republic). Egg albumin used for the detection of anti-inflammatory action was provided by eggs purchased from the local market.

### 2.2 Plant material

Plant materials (leaves, stems, and flowers) were collected in the blooming stage in June 2023 from Polyrrachos village (at 40°08′23.0″N and 21°57'12.0″E, based on Google Earth version 9.185.0.0) in Kozani, Municipality of Western Macedonia Greece, at about 630 m altitude. The microclimate of the area is characterized by warm summers, cool autumns and springs, and cold winters, wetter than other regions of the municipality affected by the Polyphytos Lake nearby.

After collection, the aerial parts of *S. iva* were weighed and dried for 30 days at room temperature in a shaded room without excessed humidity until their weight stabilized, indicating complete loss of water, and were subjected to extraction processes. The aerial parts were ground after drying, and the particle size of the dried plant material was determined.

### 2.3 Plant extraction with response surface methodology (RSM)

In order to maximize the efficiency of extracting polyphenols and assessing antioxidant and anti-inflammatory activity from medicinal plant extracts, the RSM technique was utilized. First, the optimal temperature and extraction time were studied. To do this, 0.4 g of the *S. iva* plant was accurately weighed (Kern PLS 3100-2F, Kern & Sohn GmbH, Balingen, Germany) using 20 mL of deionized water. As shown in Table 1, the experiments were conducted in a temperature range of 20–80°C and the time of extraction ranged from 30 to 90 min. The experiment that used a Main Effects Screening design with a major impact screening arrangement served as the basis for the optimization procedure. There were 10 design points in the experiment. The experimental design resulted in the creation of five levels of process variables. Analysis of variance (ANOVA) and summary-of-fit tests were used to evaluate the overall model significance, as indicated by the *R* ^2^ and *p*-values, and the significance of the model coefficient, as indicated by the equation, with a minimum level of 95% confidence. After the 500-rpm stirring extraction process was finished, the samples were centrifuged at 10,000 × *g* for 10 min at room temperature using a NEYA 16R Remi Elektrotechnik Ltd. (Palghar, India). The supernatants were then gathered and kept in storage at −40°C.

**Table 1:** Actual and coded levels of the independent variables were used to optimize the extraction process using the Main Effects Screening design

Independent variables	Code units	Coded variable level				
		1	2	3	4	5
Temperature (*T*, °C)	*X*_1_	20	35	50	65	80
Time (*t*, min)	*X*_2_	30	45	60	75	90

Additionally, a quadratic (second-order) polynomial model was used to predict the response variable as a function of the investigated independent components, as shown by equation (1):

(1) \[{Y}_{k}\text{}=\text{}{\beta }_{0}+\mathop{\sum }\limits_{i=1}^{2}{\beta }_{i}{X}_{i}+\mathop{\sum }\limits_{i=1}^{2}{\beta }_{{ii}}{\text{X}}_{i}^{2}+\mathop{\sum }\limits_{i=1}^{2}\mathop{\sum }\limits_{j=i+1}^{3}{\beta }_{{ij}}{X}_{i}{X}_{j}.]\]

The predicted response variable is denoted as *Y*_*k*_, while the independent variables are *X*_*i*_ and *X*_*j*_. The intercept and regression coefficients for the linear, quadratic, and interaction terms of the model are denoted as *β*_0_, *β*_*i*_, *β*_*ii*_, and *β*_*ij*_, respectively.

The RSM was used to identify the largest peak region and evaluate the impact of a significant independent variable on the response [29]. To visually depict the model equation, the creation of a three-dimensional surface response graph took place.

### 2.4 Other extraction techniques

In order to confirm that water is the optimal extraction solvent, other solvents were used for extraction purposes, such as ethanol as an alternative solvent. Specifically, 2 g of the dried *S. iva* sample were extracted in 100 mL ethanol as well as in ethanol with water mixture (60:40 v/v) at 50°C for 30 min. In contrast, two more plant samples of the same weight were extracted in deionized water at 100°C for 5 min and at 50°C for 30 min in order to collect comparable results.

### 2.5 Polyphenol determination

#### 2.5.1 Total polyphenol content (TPC)

A previously established methodology [30] was applied to determine TPC. Briefly, 100 μL of the properly diluted sample extract was mixed with 100 μL of Folin–Ciocalteu reagent, and after 2 min, 800 μL of 5% w/v aqueous sodium carbonate solution was added. The mixture was incubated at 40°C for 20 min and the absorbance was recorded at 740 nm in a Shimadzu UV-1700 PharmaSpec Spectrophotometer (Kyoto, Japan). The TPC (*C*_TP_) was calculated from a gallic acid calibration curve (10–80 mg/L). TPC was determined as mg gallic acid equivalents (GAE) per g of dry weight (dw), using the following equation:

(2) \[\text{TPC}(\text{mg GAE}\left/\text{g dw})\text{}=\text{}\frac{{C}_{{\mathrm{TP}}}\times {V}}{w},]\]

where the volume of the extraction medium is indicated with *V* (expressed in L) and the dry weight of the sample as *w* (expressed in g).

#### 2.5.2 HPLC quantification of polyphenolic compounds

HPLC was used to detect and quantify individual polyphenols from the sample extracts, as established in our previous research [30]. A Shimadzu CBM-20A liquid chromatograph and a Shimadzu SPD-M20A diode array detector (DAD) (both purchased by Shimadzu Europa GmbH, Duisburg, Germany) were employed for the analysis of medicinal plant extracts. The compounds were separated into a Phenomenex Luna C18(2) column from Phenomenex Inc. (Torrance, CA, USA), kept at 40°C (100 Å, 5 μm, 4.6 mm × 250 mm). The mobile phase included 0.5% aqueous formic acid (A) and 0.5% formic acid in acetonitrile/water (3:2) (B). The gradient program required: initially from 0 to 40% B, then to 50% B in 10 min, to 70% B in another 10 min, and then constant for 10 min. The flow rate of the mobile phase was set at 1 mL/min. The compounds were identified at 320 nm by comparing the absorbance spectrum and retention time to those of pure standards and then quantified through calibration curves (0–50 μg/mL).

### 2.6 Antioxidant capacity of the extracts

#### 2.6.1 FRAP assay

An established technique by Shehata et al. [31] was used for the evaluation of FRAP. In a 1.5-mL Eppendorf tube, 50 μL of the properly diluted sample extract was mixed with 50 μL of FeCl_3_ solution (4 mM in 0.05 M HCl). The mixture was incubated for 30 min at 37°C, with 900 μL of TPTZ solution (1 mM in 0.05 M HCl) being immediately added right after, and the absorbance was measured after 5 min at 620 nm. The ferric-reducing power (*P*_R_) was calculated using an ascorbic acid calibration curve (*C*_AA_) in 0.05 M HCl with ranging values (50–500 μM). The *P*_R_ was calculated as μmol of ascorbic acid equivalents (AAE) per g of dw, using equation (3):

(3) \[{P}_{\text{R}}\text{}(\mu\text{mol AAE}\left/\text{g d}\text{w})\text{}=\text{}\frac{{C}_{{\mathrm{AA}}}{\mathrm{\times }}V}{w},]\]

where *V* is represented (in L) as the entire volume of the extraction medium and *w* (in g) represents the dried weight of the material.

#### 2.6.2 DPPH^•^ antiradical activity assay

The extracted polyphenols from the dried material were evaluated for their antiradical activity (*A*_AR_) using a slightly modified DPPH˙ method, as previously established by Shehata et al. [31]. In brief, 25 μL of the properly diluted sample extract was mixed with a quantity of 975 μL of a 100 μM DPPH˙ solution in methanol, with the solution being kept at room temperature for 30 min in the dark right after. The absorbance was measured at 515 nm. Moreover, a blank sample was used instead of the sample, including DPPH˙ solution and methanol, with the absorbance immediately being measured. To calculate the percentage of scavenging, equation (4) was employed:

(4) \[ \% \text{Scavenging}=\frac{{A}_{\text{control}}-{A}_{\text{sample}}}{{A}_{\text{control}}}\text{}\times \text{}100.]\]

An ascorbic acid calibration curve (*C*_AA_, 100–1,000 μmol/L in methanol) in equation (5) was used to evaluate antiradical activity (*A*_AR_), which was expressed as μmol AAE per g of dw:

(5) \[{A}_{\text{AR}}\text{}(\mu\text{mol AAE}\left/\text{g d}\text{w})\text{}=\text{}\frac{{C}_{{\mathrm{AA}}}\times V}{w},]\]

where *V* is represented (in L) as the entire volume of the extraction medium and *w* (in g) represents the dried weight of the material.

#### 2.6.3 Hydrogen peroxide (H_2_O_2_) scavenging assay

A previous method [32] was applied for the H_2_O_2_ scavenging assay. A quantity of 400 μL of the properly diluted sample extract and 600 μL of an H_2_O_2_ solution (40 mM, made in phosphate buffer, pH 7.4) was added into an Eppendorf tube. The absorbance was recorded right after 10 min at 230 nm. The scavenging capacity of the H_2_O_2_ was expressed as follows:

(6) \[ \% \text{Scavenging of}{\text{H}}_{2}{\text{O}}_{2}\text{}=\text{}\frac{{A}_{0}\left-\left({A-A}_{\text{c}})}{{A}_{0}}\text{}\times \text{}100,]\]

where the absorbances of the blank solution, the extract solution in the absence of hydrogen peroxide, and the sample are denoted by *A*_0_, *A*_c_, and *A*, respectively.

The concentration of ascorbic acid ranged in the calibration curve (*C*_AA_, 50–500 μmol/L in 0.05 M HCl) and the following equation (7) was used to determine the anti-hydrogen peroxide activity (*A*_AHP_) as μmol AAE per g of dw:

(7) \[{A}_{\text{AHP}}\text{}(\mu\text{mol AAE}\left/\text{g d}\text{w})\text{}=\text{}\frac{{C}_{{\mathrm{AA}}}\times V}{w},]\]

where *V* denotes the volume of the extraction medium (in L), and *w* is the dry weight of the sample.

### 2.7 Assessment of *in vitro* anti-inflammatory activity

The *in vitro* evaluation of the anti-inflammatory properties of the *S. iva* extracts was conducted using the albumin denaturation assay [33]. Briefly, a mixture containing egg albumin and PBS (pH = 6.4) with a 0.1:1.4 mL ratio, or 6.67% v/v (mixture A), respectively, was prepared. Then, 400 μL of the properly diluted sample extract or standard were mixed with 600 μL of mixture A in a 1.5 mL Eppendorf tube, and then, the tubes were incubated at 37°C for 15 min. Afterwards, the mixture was heated at 70°C for 5 min. The absorbance was then recorded at 660 nm. This wavelength was chosen to minimize interference from the extract’s color or turbidity. To determine the % inhibition of protein denaturation, the following equation (8) was used:

(8) \[ \% \text{Inhibition}=\left|\frac{{\text{Absorbance}}_{\text{sample}}}{{\text{Absorbance}}_{\text{control}}}-\text{}1\right|\times \text{}100.]\]

By measuring the absorbance of the control (without extract) against that of the sample (with extract), we can assess the degree to which the extract prevents protein denaturation.

### 2.8 Statistical analysis

The statistical analysis was applied to the RSM through Main Effects Screening design and distribution analysis, using the JMP^®^ Pro 16 program (SAS, Cary, NC, USA). The extraction processes were carried out at least twice for every batch of plant extract, and the quantitative analysis was carried out in triplicate. The Kolmogorov–Smirnov test was employed to verify data normality. To detect statistically significant differences, a one-way ANOVA was conducted, succeeded by a post hoc Tukey HSD (honestly significant difference) test using the Tukey–Kramer method. The means and standard deviations of the results are used to demonstrate them. Using JMP^®^ Pro 16 software, Pareto plot analysis, principal component analysis (PCA), and multivariate correlation analysis (MCA) were carried out.

## 3 Results and discussion

### 3.1 Optimization of extraction conditions

Even though extraction is considered to be a common laboratory technique, it may result in being energy consuming, requiring also a significant amount of solvents, as each plant has a divergent abundance of bioactive compounds with different chemical properties. Supposing that cost-effectiveness is not taken into consideration, the majority of the organic solvents being used are harmful to the environment. Moreover, it may be necessary for the plant material to be extracted with many different solvents in order to conclude which of them and under which conditions is able to extract as many substances as possible, providing the researcher with an extraction that may lead to significant conclusions about the plant’s potential properties [34]. Additionally, the extraction duration and temperature are crucial parameters that define the energy consumption during the procedure. Obviously, the detection of optimal extraction conditions is necessary as it enhances the efficiency of the procedure, and utilizing water as solvent seems to be the most suitable choice, as it is approachable, affordable, and without environmental impact [35]. Investigating the effect of temperature and duration on the extraction process has improved our understanding of optimizing the yield and efficacy of polyphenolic compounds. This knowledge is crucial for devising more effective extraction techniques that ensure the preservation and maximization of bioactive compounds.

**Figure 2:**
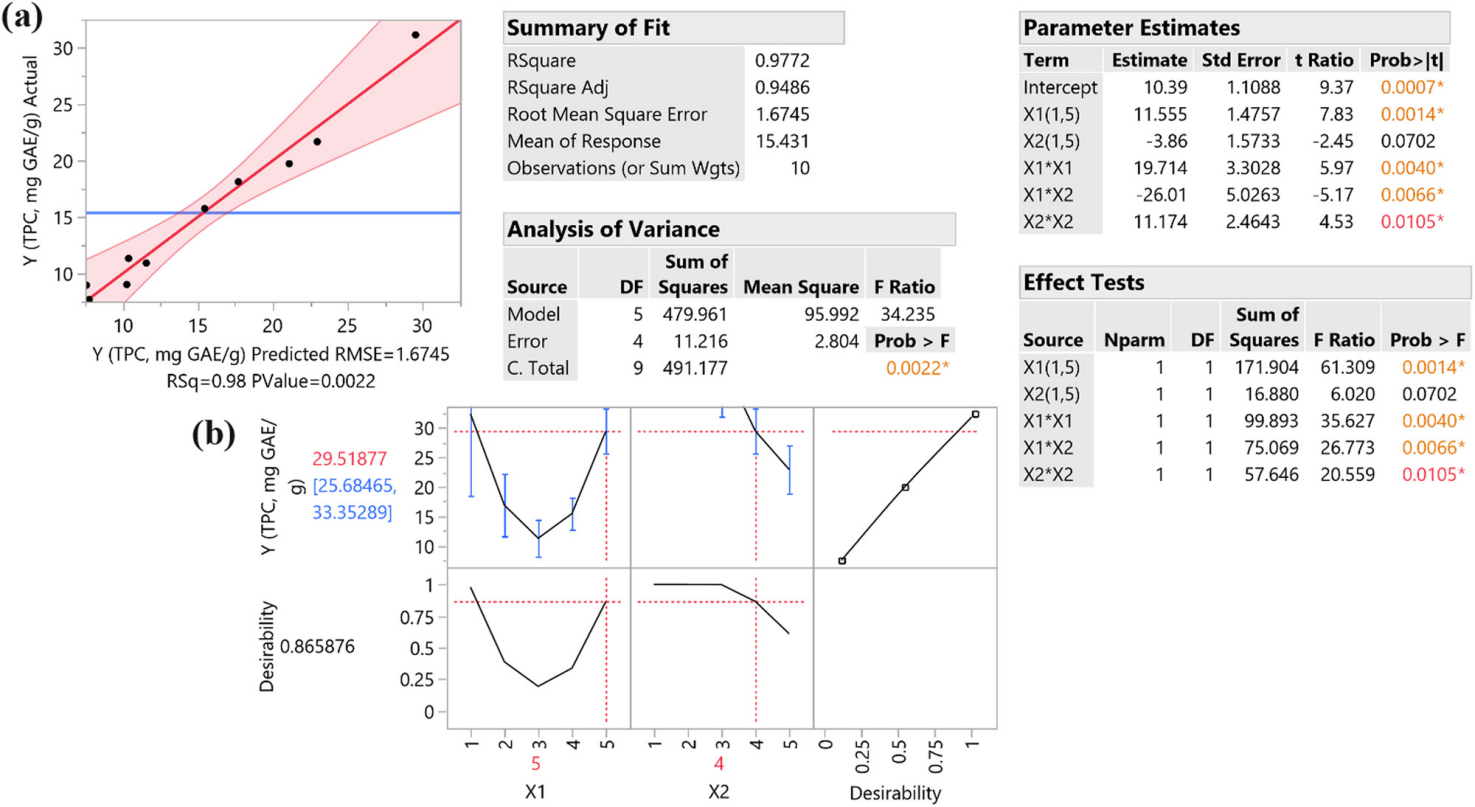
Plot (a) displays the actual versus the predicted response (TPC, mg GAE/g dw) for the optimization of extraction of *S. iva* plant performed with water solutions. The inset tables provide statistics related to the evaluation of the resulting model. Values with color and asterisk are statistically significant. The desirability function for the optimization of extraction of *S. iva* performed with water solutions is displayed in Plot (b).

**Figure 3:**
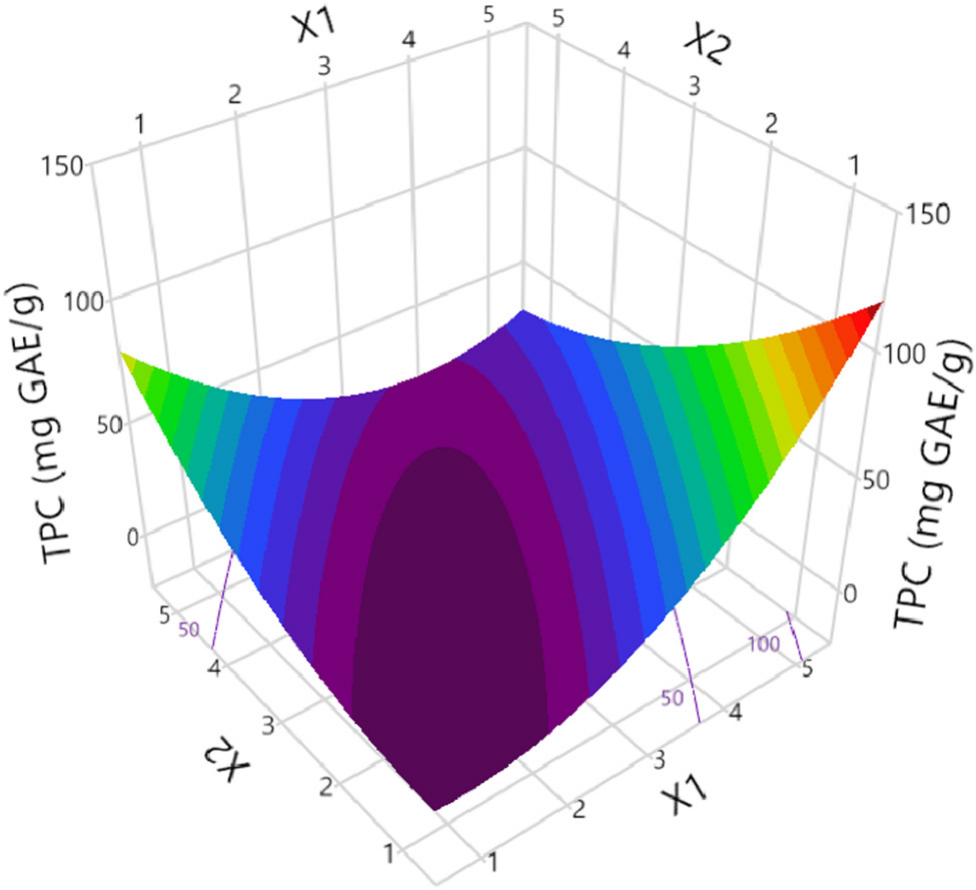
Three-dimensional graph depicting the covariation of *X*_1_ (*T*, °C) and *X*_2_ (*t*, min) and the effect of the process variables considered on the response (TPC, mg GAE/g dw) for the optimization of extraction of *S. iva* performed in water solutions.

Taking all the above into consideration, as well as the predicted antioxidant activity and TPC of the extract, we came up with a correlation between the extraction conditions and the extract’s properties using the RSM approach, as presented in Tables 2 and 3, as an attempt to investigate the optimal design point. Obviously, extracting the plant material for 75 min at 80°C (design point-DP 5) seems to provide us with an extract rich in polyphenolic compounds and with enhanced antioxidant potency, as it is demonstrated in Figure 2, where the predicted response referring to parameters of DP 5 presenting as statistically significant. The optimal temperature was quite anticipated, as there are references documenting that the highest polyphenolic substance recovery is conducted under 50–80°C [36]. Also, the three-dimensional plot for TPC can be seen in Figure 3.

**Table 2:** Experimental findings for the two independent variables under investigation and the dependent variable’s response to TPC

Design point	Independent variables		Response TPC (mg GAE/g dw)	
	*X*_1_ (*T*, °C)	*X*_2_ (*t*, min)	Actual	Predicted
1	1 (20)	2 (45)	9.02 ± 0.62	10.27
2	2 (35)	2 (45)	7.69 ± 0.43	7.76
3	3 (50)	3 (60)	11.33 ± 0.56	10.39
4	4 (65)	4 (75)	15.75 ± 1.04	15.46
5	5 (80)	4 (75)	31.13 ± 2.18	29.52
6	1 (20)	1 (30)	8.96 ± 0.34	7.58
7	2 (35)	1 (30)	10.92 ± 0.29	11.57
8	3 (50)	5 (90)	18.12 ± 1.20	17.70
9	4 (65)	3 (60)	19.72 ± 0.67	21.10
10	5 (80)	5 (90)	21.67 ± 1.13	22.97

**Table 3:** Coded values of the two independent variables under investigation and the actual values of antioxidant and anti-inflammatory activity assays

Design point	Independent variables		Responses			
	*X*_1_ (*T*, °C)	*X*_2_ (*t*, min)	FRAP (μmol AAE/g dw)	DPPH (μmol AAE/g dw)	Anti-hydrogen peroxide activity (μmol AAE/g dw)	Anti-inflammatory activity (%)
1	1 (20)	2 (45)	47.02 ± 3.53	17.81 ± 1.26	129.78 ± 5.84	42.53 ± 2.64
2	2 (35)	2 (45)	41.55 ± 2.99	14.03 ± 0.86	76.86 ± 1.77	42.30 ± 3.13
3	3 (50)	3 (60)	80.05 ± 1.68	32.92 ± 1.35	192.42 ± 14.24	44.50 ± 2.18
4	4 (65)	4 (75)	94.69 ± 5.40	59.18 ± 3.08	301.86 ± 14.79	56.02 ± 3.53
5	5 (80)	4 (75)	182.85 ± 11.15	95.83 ± 5.17	459.36 ± 31.70	72.53 ± 4.71
6	1 (20)	1 (30)	51.04 ± 3.67	16.29 ± 0.46	82.44 ± 3.38	47.61 ± 3.52
7	2 (35)	1 (30)	60.73 ± 3.16	22.41 ± 1.43	127.80 ± 6.77	55.69 ± 1.28
8	3 (50)	5 (90)	98.50 ± 3.25	47.72 ± 2.72	270.18 ± 7.57	55.09 ± 3.97
9	4 (65)	3 (60)	107.31 ± 3.97	49.69 ± 1.94	280.98 ± 16.02	60.83 ± 3.35
10	5 (80)	5 (90)	131.41 ± 6.44	58.88 ± 3.36	348.84 ± 10.12	64.17 ± 4.17

The desirability function was used in the optimization process to combine multiple response variables into a single composite response. Each response variable was transformed into a desirability value ranging from 0 (completely undesirable) to 1 (fully desirable). The overall desirability was calculated as the geometric mean of the individual desirability values, providing a single metric that reflects the optimal conditions for all response variables. This approach allows for the simultaneous optimization of multiple criteria, ensuring a balanced and comprehensive evaluation of the extraction conditions.

The TPC was expressed in milligrams of gallic acid equivalents per gram of dry weight (mg GAE/g dw). This unit standardizes the phenolic content based on a gallic acid calibration curve, allowing for comparison across different studies and samples. The antioxidant capacity measured by assays such as FRAP and DPPH was expressed in micromoles of ascorbic acid equivalents per gram of dry weight (µmol AAE/g dw). This unit standardizes the antioxidant activity based on an ascorbic acid calibration curve.

The ferric-reducing antioxidant power (FRAP) assay, commonly utilized to assess the antioxidant capacity of different samples, presents multiple limitations. Its high sensitivity to pH fluctuations, especially it is performed at an acidic pH of approximately 3.6, may not provide an accurate measure of antioxidant capacity at physiological pH. Moreover, temperature changes can affect the outcomes, with elevated temperatures possibly causing an overestimation and reduced temperatures an underestimation. The FRAP assay is designed to measure the reducing power of antioxidants, which may not reflect all antioxidant activities, such as radical scavenging or metal chelation. This limitation restricts its capacity for a complete assessment. Additionally, the assay can be affected by other reducing agents present in the sample, potentially leading to erroneous outcomes. Nevertheless, the FRAP assay is valued for its simplicity, rapidity, cost-efficiency, and consistent reproducibility when standard conditions are applied. Ascorbic acid is often employed as a benchmark for its notable antioxidant properties; however, it might not adequately reflect the intricate antioxidant activities of plant extracts. Employing alternative methods, such as a blend of standards like Trolox, or integrating the FRAP assay with other antioxidant evaluations (for instance, DPPH, ABTS, ORAC) could offer a more comprehensive evaluation of antioxidant potential by encompassing various mechanisms of action.

The second-order polynomial equations obtained for the responses were expressed as follows:

(9) \[\text{TPC}=9.83-4.29{X}_{1}+0.82{X}_{2}+4.93{X}_{1}^{2}+2.79{X}_{2}^{2}-6.5{X}_{1}{X}_{2},]\]

(10) \[\text{FRAP}=50.19-19.58{X}_{1}+5.14{X}_{2}+22.88{X}_{1}^{2}+11.88{X}_{2}^{2}-28.27{X}_{1}{X}_{2},]\]

(11) \[\text{DPPH}=6.75-8.96{X}_{1}+10.54{X}_{2}+11.95{X}_{1}^{2}+5.28{X}_{2}^{2}-15.06{X}_{1}{X}_{2},]\]

(12) \[\text{Anti-hydrogen peroxide activity}\hspace{1em}=49.87-47.11{X}_{1}+56.22{X}_{2}+54.34{X}_{1}^{2}+23.84{X}_{2}^{2}-66.99{X}_{1}{X}_{2},]\]

(13) \[\text{Anti-inflammatory activity}=55.25-0.23{X}_{1}-10.95{X}_{2}+5.75{X}_{1}^{2}+4.99{X}_{2}^{2}-8.09{X}_{1}{X}_{2},]\]

Depending on ANOVA analysis results, we may also evaluate the responses of the extract, as an attempt to assess its potent properties. Apparently, all the responses of the conducted experiments seem to be statistically significant, indicating the pharmacological properties of *S. iva* plant extract. Moreover, according to the RMSE, total phenolic content and anti-inflammatory activity appear to be closer to the actual values, so we anticipated these properties to be experimentally proved, as demonstrated in Table 4.

**Table 4:** ANOVA analysis of the responses

Responses	*F*-ratio	*p*-value	*R* ^2^	Adjusted *R* ^2^	RMSE
TPC	34.24	0.0022	0.9772	0.9486	1.67
FRAP	15.65	0.0099	0.9514	0.8906	14.54
DPPH	8.57	0.0292	0.9146	0.8078	11.38
Anti-hydrogen peroxide activity	22.03	0.0052	0.9650	0.9212	35.41
Anti-inflammatory activity	46.11	0.0013	0.9829	0.9616	1.96

RMSE means root mean square error.

### 3.2 Impact of extraction parameters on assays through Pareto plot analysis

**Figure 4:**
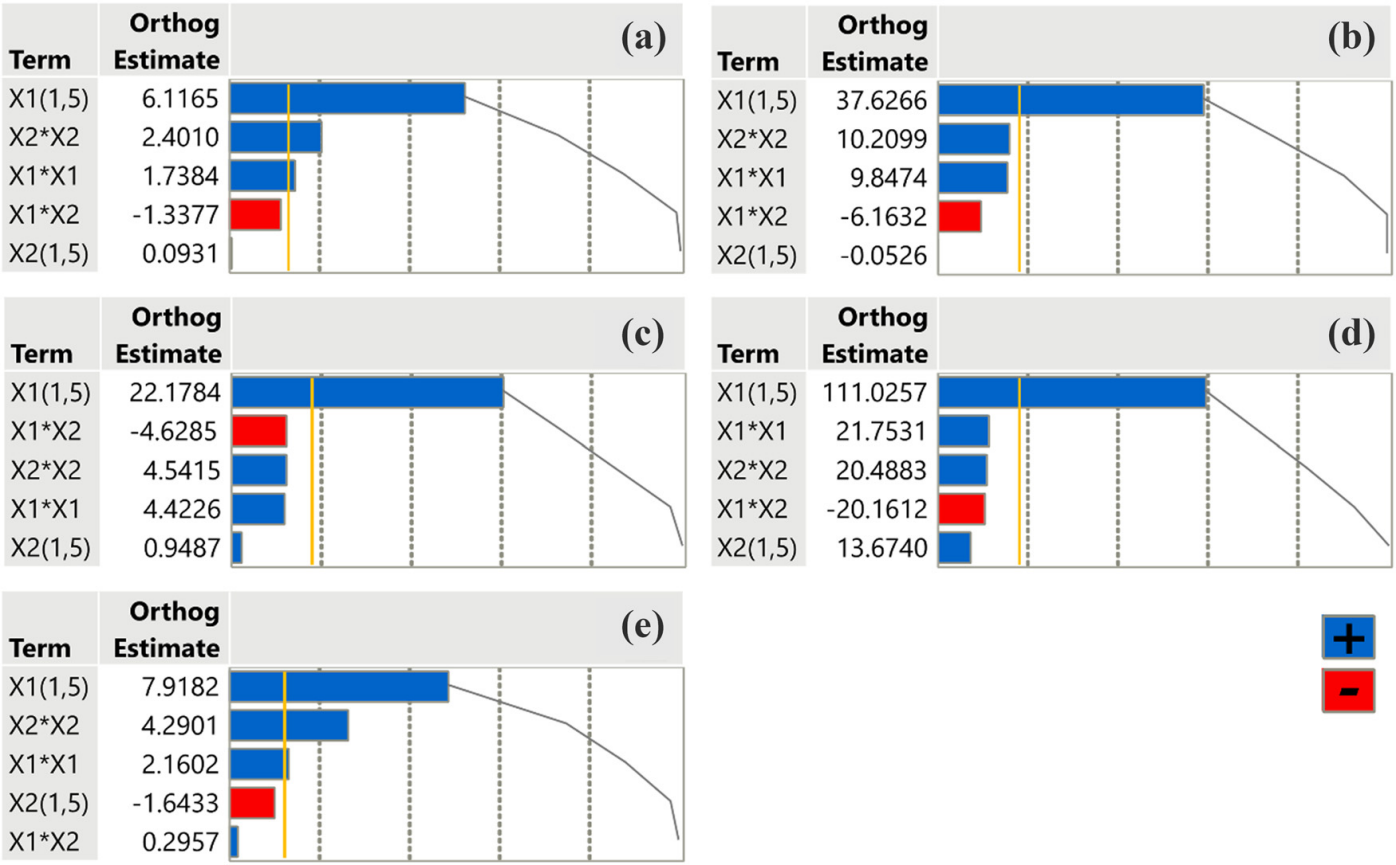
Pareto plots of transformed estimates for TPC (a), FRAP (b), DPPH (c), anti-hydrogen peroxide activity (d), and anti-inflammatory activity (e) assays. A reference gold line is drawn on the plot to indicate the level of significance (*p* < 0.05).

In Pareto plot analysis, factors are chosen for their contribution to total variance, with the most influential ones accounting for the largest percentage of variance. In order to examine the positive or negative impact of extraction parameters on the extract properties, we conducted a Pareto plot analysis including results from all the experimental assays that took place. Observing the plots (Figure 4), we may conclude to the fact that the combination of temperature and time of extraction does not affect either the TPC of the extract or its antioxidant and anti-inflammatory properties, as it is demonstrated to adopt negative or very low values. On the other hand, the temperature itself (factor *X*_1_) mainly contributes to eventually collecting the optimal extract. The temperature not only affects the extract’s quality but also overcomes the critical value’s threshold (gold line) indicating statistical significance. Pareto plot analysis may occasionally simplify the complex interplay between factors too much, which could lead to a misinterpretation of the results.

### 3.3 Optimal extraction conditions

**Figure 5:**
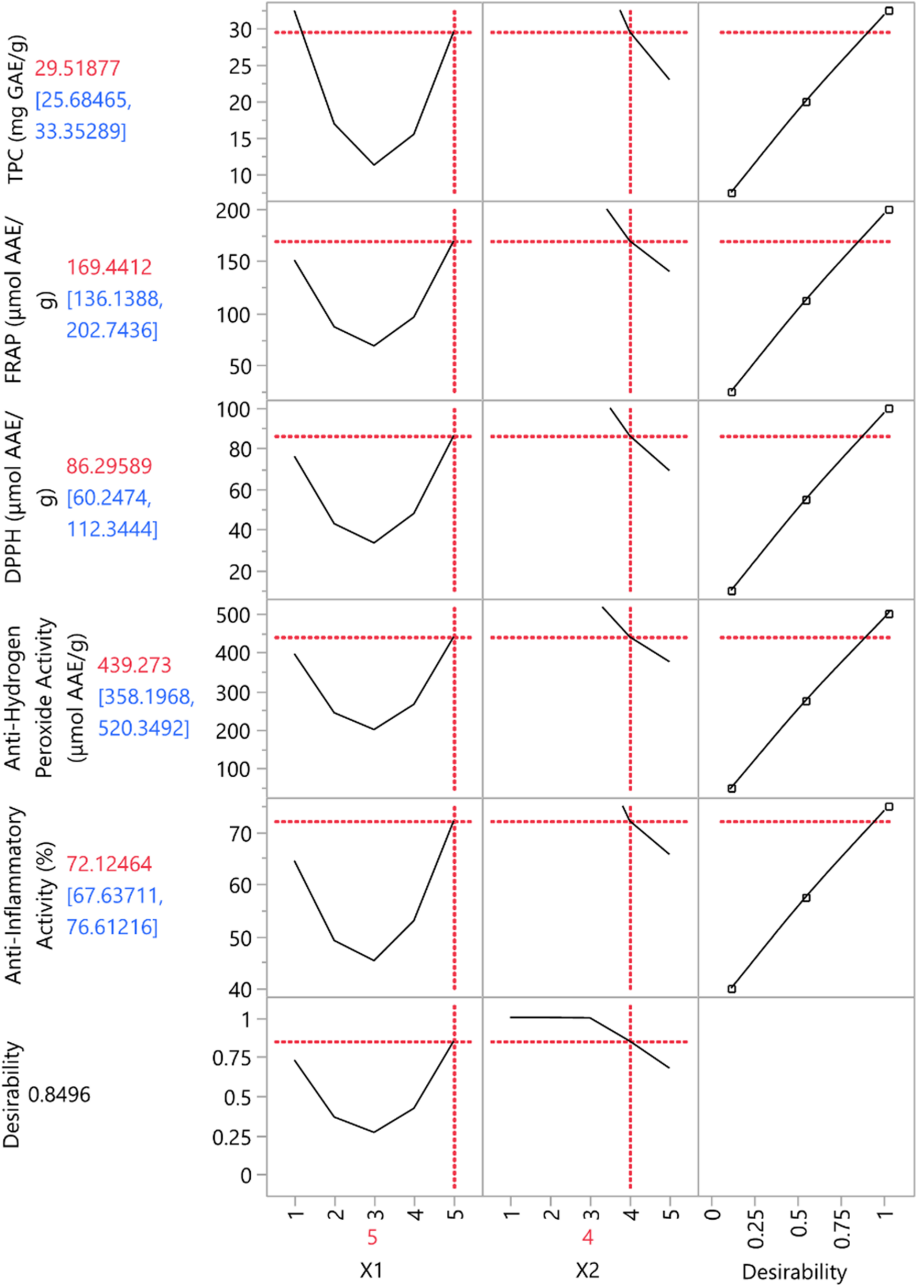
Maximum predicted responses and optimum extraction conditions for the dependent variables.

Attempting to optimize the extraction conditions furthermore, partial least squares (PLS) analysis was used for the assessment of the extraction parameters impact, as depicted in Figure 5. Taking into consideration that desirability equals 0.8496, variables estimated over this point seem to have a significant contribution. As demonstrated in the plot, temperature (*X*_1_ variable) affects the quality of extract, the quantity of polyphenols (TPC), and its antioxidant and anti-inflammatory properties. Specifically, the DP5 (80°C) seems to provide the optimum results. Examining the impact of extraction duration (*X*_2_ variable), it can be noticed that this parameter solely does not determine the extract’s content at the same level comparatively with the temperature. However, extracting *S. iva* at 80°C for 75 min (DP5) is predicted to be the ideal parameter combination.

### 3.4 PCA and MCA

**Figure 6:**
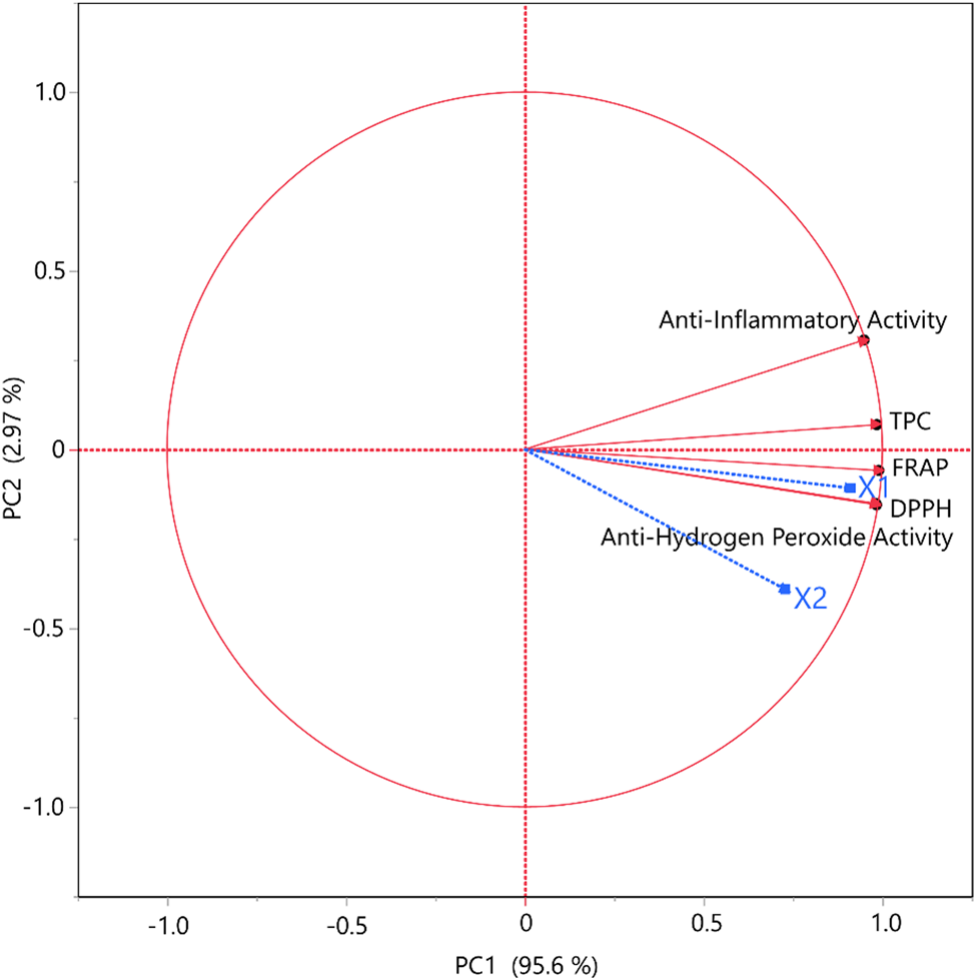
PCA for the measured variables. Each *X* variable is presented with a blue color.

To obtain a more objective aspect of how the temperature and the duration of the extraction are correlated to the TPC, the antioxidant (FRAP, DPPH, H_2_O_2_), and anti-inflammatory assays, a PCA was conducted. In PCA, the selection criteria focus on identifying the principal components that account for the majority of the variance in the data, those with eigenvalues >1. As the parameter’s sign determines whether or not two or more variables are associated, we may conclude once again, according to Figure 6, that the extraction’s temperature positively affects the extract’s properties and its concentration of polyphenolic compounds. Additionally, all the variables were well discriminated, and it was revealed that TPC and FRAP assay, are also positively correlated. On the contrary, the negative sign of extraction’s duration, reveals, as anticipated, that this parameter does not noticeably determine the extract’s composition or its experimental assays measurements. PCA is beneficial for dimensionality reduction but may not consistently capture the non-linear relationships between variables.

Multiple correspondence analysis was utilized to discern correlations among various variables, with the most significant factors identified by the highest correlation coefficients. Moreover, taking into consideration the data displayed in Table 5, the high association among all the parameters is also confirmed as they all have positive signs and correlation >0.89. A low correlation, compared to other measured variable pairs, was shown between anti-inflammatory activity and anti-hydrogen peroxide activity (0.8866), in contrast with the highest correlation that appears among the latter and DPPH (0.9858). MCA may be constrained by multicollinearity and the possibility of spurious correlations.

**Table 5:** Multivariate correlation analysis of measured variables

Responses	TPC	FRAP	DPPH	Anti-hydrogen peroxide activity	Anti-inflammatory activity
TPC	—	0.9691	0.9411	0.9583	0.9419
FRAP		—	0.9755	0.9740	0.9177
DPPH			—	0.9858	0.8924
Anti-hydrogen peroxide activity				—	0.8866
Anti-inflammatory activity					—

### 3.5 Optimal extract and comparison with other extraction techniques

The correlation between the values generated by the second-order polynomial model and those acquired through experimental analysis is determined to be 0.9973, and they show no deviations with the *p*-value being 0.0002. Comparing data from Table 6, the optimal extract’s responses are similar to the maximum predicted ones and mainly seem more increased than the latter, concluding that extracting at 80°C for 75 min is the ideal condition for *S. iva.* Extracting in boiling water for 5 min and in water:ethanol mixture (40:60 v/v) at 50°C for 30 min, leads to quite similar and pretty significant experimental responses, except the TPC, but lower than the expecting ones. This mixture was chosen based on its ability to extract a broad range of polyphenolic compounds. The less interesting results that depict significantly decreased compared to the optimal extract are derived if we extract the plant material with pure ethanol or deionized water at 50°C for 30 min. Ethanol was chosen as an alternative solvent due to its effectiveness in extracting polyphenolic compounds and its safety for use in food and pharmaceutical applications.

**Table 6:** Maximum predicted responses, optimal extract under optimal extraction conditions, and comparison with other extraction techniques

Extracts	Responses				
	TPC (mg GAE/g)	FRAP (μmol AAE/g)	DPPH (μmol AAE/g)	Anti-hydrogen peroxide activity (μmol AAE/g)	Anti-inflammatory activity (%)
Maximum predicted response	29.52 ± 3.83^a^	169.44 ± 33.30^a^	86.30 ± 26.05^a^	439.27 ± 81.08^a^	72.12 ± 4.49^a^
Optimal extract	31.55 ± 0.79^a^	185.78 ± 5.57^a^	95.40 ± 3.91^a^	410.42 ± 16.42^a^	70.06 ± 1.47^a,b^
Boiling water	12.50 ± 0.89^c^	92.92 ± 2.23^b^	73.56 ± 4.56^a,b^	229.14 ± 16.50^b^	61.74 ± 4.01^b^
Ethanol 60% v/v	19.99 ± 0.54^b^	92.88 ± 3.81^b^	51.74 ± 3.88^b^	231.84 ± 4.64^b^	na
Ethanol	2.52 ± 0.07^d^	11.33 ± 0.69^c^	3.46 ± 0.13^c^	26.64 ± 0.80^c^	na
Water	10.99 ± 0.31^c^	32.93 ± 1.75^c^	19.80 ± 1.05^c^	63.36 ± 1.96^c^	49.57 ± 3.32^c^

Within each column, statistically significant differences (*p* < 0.05) are denoted with lowercase letters (e.g., a–d); na means not analyzed.

### 3.6 Analysis of the optimal extract

The experimental assays we conducted, indicated that *S. iva* extract is abundant in polyphenolic compounds, as we anticipated due to statistical predictions and bibliographic references to other *Stachys* species. Especially the optimal extract has the ultimate TPC, 31.55 ± 0.79 mg GAE/g dw (actual) and 29.52 ± 3.83 mg GAE/g dw (predicted response), compared to others. The polyphenol compounds as we mentioned are mainly responsible for the plant’s beneficial properties, affecting its antioxidant and anti-inflammatory activity. This is also proved by the fact that all these variables correlate and their values are associated, as results from our data analysis.

**Figure 7:**
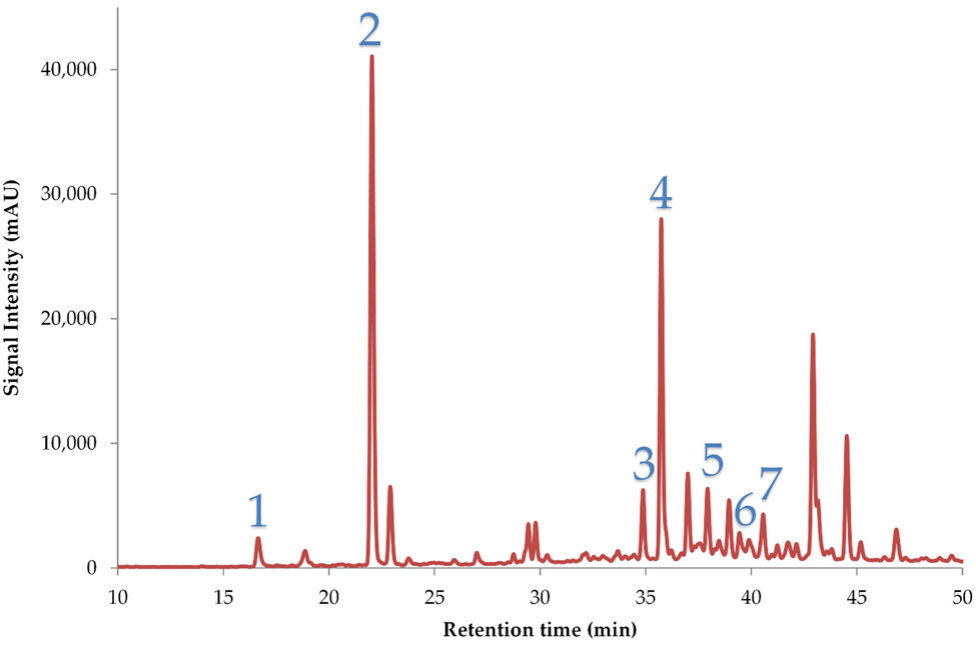
Representative HPLC chromatogram at 320 nm of optimal extract, demonstrating polyphenolic compounds that were identified. 1: Neochlorogenic acid; 2: chlorogenic acid; 3: rutin; 4: verbascoside; 5: narirutin; 6: myricetin; 7: rosmarinic acid.

The main polyphenolic compounds of the optimal extract were identified and also quantified, data demonstrated in Table 7, by using HPLC-DAD. A representative chromatogram is illustrated in Figure 7, where according to the retention time of each compound and their UV spectrum, phenolic acids such as rosmarinic, neochlorogenic and chlorogenic acid, and flavonoids (rutin, verbascoside, narirutin, myricetin) were identified.

**Table 7:** Polyphenolic compounds analysis of optimal extract under optimal extraction conditions

Polyphenolic compound	Optimal extract (mg/g dw)
Neochlorogenic acid	0.55 ± 0.03
Chlorogenic acid	5.02 ± 0.21
Rutin	0.45 ± 0.02
Verbascoside	0.87 ± 0.06
Narirutin	0.03 ± 0
Myricetin	0.46 ± 0.01
Rosmarinic acid	1.4 ± 0.04
Total identified	8.79 ± 0.38

Examining further each compound’s properties we may better understand the traditional use of “fluffy mountain tea’s” extract as a remedy for curing a wide range of health conditions. Initially, referring to rosmarinic acid (RA), many studies have been conducted both *in vivo* and *in vitro*, trying to suggest its mechanisms of action against inflammation and oxidative stress. RA’s chemical structure allows the compound to be lipophilic enough to penetrate the cell membrane and protect the cell by scavenging free radicals. Its antioxidant properties protect different organs such as liver and heart enhancing also its potent use as a neuroprotective and anticancer agent. Furthermore, studies are reporting RA’s action against different types of bacteria and fungi as well as against inflammatory conditions, possibly by inhibiting the synthesis of cytokines and suppresses the activation of cyclooxygenase and even of T-leukocytes in autoimmune diseases [37,38,39,40].

Another well-known phenolic acid that is pretty ubiquitous in plant extracts, especially in coffee and tea infusions, and with the highest concentration in our extract is chlorogenic acid (CGA). Despite its action against free radicals and inflammation due to its chemical properties, CGA seems to affect glucose absorption decreasing its concentration in blood circulation. Thus, fat burning increases as an alternative source of energy, leading to both antidiabetic and anti-obesity action. Additionally, clinical trials proved that CGA consumption is associated with attenuation of blood pressure, probably because of the induction of vessel relaxation, antimicrobial activity has also been reported [41,42,43]. As for neochlorogenic acid, that is CGA’s isomer and has similar properties. However, we may highlight that its anti-inflammatory activity derives from the inhibition of lipopolysaccharide (LPS). The latter acts like a stimulant that activates microglia (type of macrophages), causing neuroinflammation and triggering the onset of neurodegenerative diseases [44,45].

Even though phenolic acids concentration in the extract was 6.97 ± 0.28 mg/g dw out of the 8.79 ± 0.38 mg/g dw of totally identified polyphenolic compounds, many flavonoids contribute to *S. iva* properties and verbascoside (VERB) is the dominant one (0.85 ± 0.06 mg/g dw). VERB is a phenylethanoid glycoside, initially isolated from *Verbascum* sp. (mullein) but distributed in plenty of other plant species that have also been used as traditional remedies. Studies report that VERB combines anti-inflammatory, antibacterial, and even anti-androgen properties being a potent alternative approach for acne vulgaris treatment. Moreover, preventing lipoprotein oxidation protects cells from oxidative stress while repairs DNA damage caused by the latter. It may also be used as an anti-depressant, acting by triggering monoamine neurotransmitters’ production and increasing their levels and availability, while it seems to appear a protective role against atherosclerosis [46,47,48].

Rutin and myricetin are detected in approximately equal concentrations. They both possess diverse beneficial properties acting *in vitro*, as the majority of polyphenols, against lipid peroxidation, inflammation, and as radical scavengers [49]. Furthermore, rutin appears to have vasoprotective, anti-hypercholesterolemic, anti-hypertensive, and antiplatelet aggregation effects *in vitro* and after being administered to rats, while also promoting cancer cell apoptosis as indicated in experiments conducted using cell lines from various cancer types [50,51,52,53]. Highlighting some distinct properties of myricetin, we should mention that there are pieces of evidence about its intervention in diabetes and obesity management in rats [54,55].

Last but not least, even in minimum concentration, the flavonoid narirutin, which is mainly found in citrus fruit, has a plethora of beneficial properties similar to the ones documented for the rest of the extract’s flavonoids [56,57,58,59].

## 4 Conclusions

The present study aimed to identify the ideal extraction solvent (deionized water and ethanol) and optimal extraction parameters (temperature and duration) to obtain the most enriched extract of *Stachys iva*, with high therapeutical value. *S. iva* has been traditionally used as a remedy with diverse pharmacological properties, which this study attempted to validate experimentally. RSM was utilized to identify the optimal extraction conditions, combined with Pareto plot analysis. Additionally, PCA and MCA detected positive correlations between experimental assay responses and extraction conditions, as well as the association among these responses.

Each extract underwent experimental procedures to determine its TPC, antioxidant capacity (FRAP, DPPH, anti-hydrogen peroxide activity), and anti-inflammatory activity. The collected data were compared with predicted values. HPLC analysis identified and quantified polyphenolic compounds, with a total concentration of 8.79 ± 0.38 mg/g dw. Chlorogenic acid was the dominant compound (5.02 ± 0.21 mg/g dw), followed by verbascoside (0.87 ± 0.06 mg/g dw), neochlorogenic acid, rutin, narirutin, myricetin, and rosmarinic acid.

Considering evidence from *in vitro*/*in vivo* studies and clinical trials referring to these compounds’ beneficial properties, the traditional use of *S. iva* extracts as antioxidant, anti-inflammatory, antimicrobial, and antidiabetic agents can be experimentally validated. These compounds also show potential pharmacological properties against neurodegenerative and other diseases, leading to further research on *S. iva* with promising results.

Future research will explore the use of alternative solvents, such as ethanol, methanol, and acetone, which may enhance the extraction efficiency of bioactive compounds from *S. iva*. The selection of solvents will be based on their polarity, safety, and environmental impact. Additional response variables such as flavonoid content, tannin content, and specific antioxidant activities (e.g., superoxide dismutase activity) will be investigated to provide a more comprehensive understanding of the bioactive profile of *S. iva* extracts. Advanced extraction techniques such as supercritical fluid extraction, microwave-assisted extraction, and ultrasound-assisted extraction will be explored to optimize the yield and quality of bioactive compounds. These techniques offer potential benefits in terms of efficiency, selectivity, and environmental sustainability.

Moreover, the investigation of *S. iva’s* composition of bioactive compounds and the experimental justification of its traditional uses may lead to a well-established use of this herb in products of food, cosmetic, and pharmaceutical industries. *S. iva* infusions may replace water or other extracts in lotions and creams, providing them with an aqueous phase rich in polyphenols and antioxidants enhancing the skin barrier against free radicals. Due to its abundance in polyphenolic substances, *S. iva* extract may be added to beverages as flavoring and perhaps as a natural preservative due to its antimicrobial potency, resulting in the production of a functional drink. As for the pharmaceutical industry, even though *S. iva* has many potential pharmacological properties (antidiabetic, antiproliferative) as mentioned, more research is required in order to ensure the lack of toxicity, severe adverse effects, or even interaction with medications. However, its extract could be included in supplements against the common cold only if the sufficiency of safety data is proven.
